# Neurosteroid Activation of GABA-A Receptors: A Potential Treatment Target for Symptoms in Primary Biliary Cholangitis?

**DOI:** 10.1155/2022/3618090

**Published:** 2022-12-06

**Authors:** Aaron Wetten, Laura Ogle, George Mells, Vinod S. Hegade, Laura Jopson, Margaret Corrigan, Jeremy Palmer, Maja Johansson, Torbjörn Bäckström, Magnus Doverskog, David E. J. Jones, Jessica K. Dyson

**Affiliations:** ^1^Translational and Clinical Research Institute, Newcastle University, Newcastle-upon-Tyne, UK; ^2^Freeman Hospital, Newcastle-upon-Tyne, UK; ^3^Department of Human Genetics, University of Cambridge, Cambridge, UK; ^4^Leeds Liver Unit, St James' University Hospital, Leeds, UK; ^5^Liverpool University Hospitals NHS Foundation Trust, Liverpool, UK; ^6^Umecrine Cognition AB, Solna, Sweden; ^7^Department of Clinical Sciences, Obstetrics and Gynecology, Umeå University, Umea, Sweden

## Abstract

**Background and Aims:**

A third of patients with primary biliary cholangitis (PBC) experience poorly understood cognitive symptoms, with a significant impact on quality of life (QOL), and no effective medical treatment. Allopregnanolone, a neurosteroid, is a positive allosteric modulator of gamma-aminobutyricacid-A (GABA-A) receptors, associated with disordered mood, cognition, and memory. This study explored associations between allopregnanolone and a disease-specific QOL scoring system (PBC-40) in PBC patients.

**Method:**

Serum allopregnanolone levels were measured in 120 phenotyped PBC patients and 40 age and gender-matched healthy controls. PBC subjects completed the PBC-40 at recruitment. Serum allopregnanolone levels were compared across PBC-40 domains for those with none/mild symptoms versus severe symptoms.

**Results:**

There were no overall differences in allopregnanolone levels between healthy controls (median = 0.03 ng/ml (IQR = 0.025)) and PBC patients (0.031 (0.42), *p* = 0.42). Within the PBC cohort, higher allopregnanolone levels were observed in younger patients (*r* (120) = −0.53, *p* < 0.001) but not healthy controls (*r* (39) = −0.21, *p* = 0.21). Allopregnanolone levels were elevated in the PBC-40 domains, cognition (*u* = 1034, *p* = 0.02), emotional (*u* = 1374, *p* = 0.004), and itch (*u* = 795, *p* = 0.03). Severe cognitive symptoms associated with a younger age: severe (50 (12)) vs. none (60 (13); *u* = 423 *p* = 0.001).

**Conclusion:**

Elevated serum allopregnanolone is associated with severe cognitive, emotional, and itch symptoms in PBC, in keeping with its known action on GABA-A receptors. Existing novel compounds targeting allopregnanolone could offer new therapies in severely symptomatic PBC, satisfying a significant unmet need.

## 1. Introduction

Primary biliary cholangitis (PBC) is a slowly progressive autoimmune liver disease resulting in obstructive cholangitis of the small intrahepatic ducts [1]; left untreated it leads to chronic cholestasis and progressive fibrosis. Fatigue and cognitive symptoms are frequently reported by patients with PBC [2, 3], and do not correlate with disease severity [2]. The PBC-40 [4], a fully validated disease-specific quality of life measure, suggests that clinically significant fatigue and/or cognitive symptoms affect over 50% and 30% of the PBC population, respectively [3, 5]. Patients presenting with symptoms are typically younger and may have more aggressive disease [6], whilst fatigue related to PBC is associated with increased all-cause mortality [7]. Recent data exploring health utility confirms that the greatest impact on functional status in PBC comes from fatigue and cognitive symptoms [8], with resultant social isolation giving rise to additional burdens and worsening quality of life [9].

Fatigue itself is complex and multi-faceted. Increasingly, it is recognised that fatigue associated with PBC has central and peripheral components, which are considered distinct pathological entities, but with subgroups of patients expressing both phenotypes [5]. Central fatigue results in “brain fog,” as a consequence of central nervous system (CNS) changes [2], whilst peripheral fatigue, “feeling like my batteries are running down,” may relate to a peripheral bioenergetic cause [10]. Cognitive symptoms in PBC, therefore, manifest in cognitive dysfunction, with difficulties relating to cognition and memory [2, 5], and are frequently associated with emotional symptoms [5]. Sleep disturbance is also common in PBC [11, 12], particularly in those with fatigue [3].

There are emerging data to suggest that even early disease-stage PBC patients with cognitive symptoms exhibit both anatomical and functional central nervous system (CNS) changes and autonomic dysfunction [13–18]. There are pronounced changes in PBC patients with cognitive symptoms [19], occurring as early as 6 months after disease diagnosis [20] with the lack of symptom improvement following liver transplantation suggesting that CNS damage may become irreversible at some point in the disease process [21–23].

Neurosteroids (synthesised in the brain from cholesterol independently of endocrine gland function) affect CNS function. Gamma-aminobutyricacid-A (GABA-A) receptors are the major biological target of the inhibitory neurotransmitter *γ*-aminobutyric acid and can be modulated by progesterone derivatives. Evidence shows them to be involved in altered sleep cycles [24], impaired cognition and memory [25, 26], fatigue in humans [27, 28], and mood disorders including premenstrual dysphoric disorder [24, 29, 30].

Allopregnanolone (3A-hydroxy-5A-pregnan-20-one), a neurosteroid and progesterone derivative, contributes to GABAergic tone [31], with elevated levels in the brain tissue of patients who died of hepatic encephalopathy (HE) [32, 33]. It has been hypothesised that fatigue related to PBC and hepatitis C (CHC) might be secondary to increased progesterone metabolites [28]. Serum allopregnanolone was found to be 80% higher in patients with PBC and CHC with significant fatigue when compared to age-matched controls, as measured using the fatigue impact scale (FIS) [34].

Cognitive symptoms remain a significant problem for many patients with PBC, with a detrimental impact on quality of life [8, 35, 36], no effective treatments [21, 37], and limited understanding of the underlying pathogenic mechanisms [38]. In this study, the relationship between PBC-associated cognitive symptoms and allopregnanolone was explored to examine whether this potentially modifiable pathway could be a target for novel therapies.

## 2. Methodology

### 2.1. Recruitment

A total of 160 serum samples were selected for allopregnanolone analysis. Of these, 120 samples were from patients with a confirmed clinical diagnosis of PBC (based on standard diagnostic criteria) [39]. This PBC cohort (comprised of 90 samples from the Birmingham and Newcastle Cohort (BANC) and 30 samples from the UK-PBC group) was phenotyped at the time of study recruitment. A further 40 healthy controls (age and gender-matched) were taken from the environmental risk factors for the aetiology of autoimmune liver disease (E-AILD). These cohorts were designed to support many diverse subprojects to explore the pathology of disease in PBC. For this pilot exploratory study, the sample size was defined by the availability of stored serum samples, with paired clinical data, in the described cohorts. All the samples used in this study had prior ethical approval for use, along with valid written informed consent obtained at the time of recruitment. This research was conducted in accordance with the International Conference on Harmonisation Good Clinical Practice Guidelines and the Declaration of Helsinki.

The BANC cohort was established as a proof-of-concept resource recruited from Queen Elizabeth Hospital, University Hospitals Birmingham NHS Foundation Trust, Birmingham, UK, and the Newcastle upon Tyne Hospitals NHS Foundation Trust, Newcastle upon Tyne, UK. It is comprised of a cross-sectional group of patients with an established diagnosis of PBC recruited between 2015 and 2017.

The UK-PBC Cohort (https://www.UK-PBC.com) is a large, prospective national cross-sectional cohort of PBC patients with detailed clinical data collection and was established to undertake studies of treatment efficacy in PBC. Within the cohort is a nested subcohort, termed the UK-PBC Nested Cohort, with additional bio-fluid sampling and banking to accompany the clinical data collection [40]. This nested cohort was designed to characterise the cellular and molecular response in PBC and facilitate the development of second-line therapies and biomarkers for more accurate stratification of treatment response and disease progression (REC ref 14/NW/1146). Recruitment was undertaken in 18 research centres, over 5 years, from 2014 to 2019.

The E-AILD cohort was established to identify disease clustering of patients within the northeast of England who had a confirmed diagnosis of autoimmune liver disease (PBC, autoimmune hepatitis, and primary sclerosing cholangitis). In conjunction, 105 healthy age and gender-matched volunteers were recruited, with optional serum samples taken. Recruitment occurred from 2015 to 2017.

All PBC patients at the point of recruitment to the relevant cohort had undertaken symptom stratification using the self-reporting PBC-40 questionnaire, which was contemporaneous with the time of serum sample collection. The PBC-40 categorises symptom severity as “none,” “mild,” “moderate” and “severe” in the following 6 domains: “cognition”; “emotional”; “itch”; “fatigue”; “social” and “general symptoms.” This score has been well described previously and the cut-offs for each domain and severity are validated [3, 4, 16] and defined in Table 1. Patients with “none” or “mild” symptoms are comparable to those found in the general population [3].

### 2.2. Allopregnanolone Assay

A liquid chromatography–tandem mass spectrometry method was developed for the analysis of allopregnanolone in human plasma or serum (Admescope, Oulu, Finland). The samples were prepared for analysis by supported liquid extraction with ethyl acetate, followed by derivatization with hydroxylamine, as previously described [41].

Standard sample spiking solutions were prepared by diluting 1 mg/ml stock solution of allopregnanolone in dimethyl sulfoxide into acetonitrile at concentrations of 2 pg/ml to 100 ng/ml. 20 *μ*l of spiking solutions were added into 180 *μ*l of active charcoal purified human serum for standard and quality control samples in a 2 ml 96-well plate. After spiking, 10 *μ*l of internal standard solution (100 ng/ml allopregnanolone-D5 in 50% methanol) was added to each sample and mixed for 5 minutes at 1000 rpm. Finally, the plate was centrifuged at 2200 g for 1 minute and 200 *μ*l of the sample was transferred onto Novum simplified liquid extraction max (Phenomenex) plate and the samples were absorbed into the plate material using a vacuum at <5 in Hg for 5–10 seconds. The plate was allowed to stand for 5 minutes (without vacuum), after which two times 500 *μ*l of ethyl acetate was allowed to flow through each well. Finally, a vacuum was applied for 20–30 seconds to allow the remaining ethyl acetate to flow through the plate. The collection plate was dried under nitrogen flow (20 L/h N2, 50°C) for 20 minutes, until dry. Finally, 150 *μ*l of 100 mM hydroxylamine in 50% methanol was added into the wells and the plate was incubated at 65°C for 60 minutes with shaking (600 rpm) to derivatize allopregnanolone into oxime-derivative for improved analytical sensitivity [41]. Finally, the plate was centrifuged for 5 minutes at 2200 g and the supernatants were transferred into a clean 1 ml 96-well plate for analysis.

Increased sensitivity was reached by the use of UniSpray ion source in a XEVO-TQ-S triple quadrupole mass spectrometer. The obtained detection limit was 0.002 ng/mL and the quantitation limit was 0.005 ng/mL, i.e., 0.006 and 0.015 nmol/L, respectively. The method accuracies in the range of 0.005 to 10 ng/mL were 91.3–107.5% (*n* = 4). Similarly, the precision (*n* = 4) was determined to be 10.5–2.5% in the concentration range of 0.005 to 10 ng/mL.

### 2.3. Statistical Analyses

Baseline characteristics of the PBC cohort, healthy controls, and serum allopregnanolone levels were analysed. This included serum allopregnanolone levels in healthy controls versus the PBC cohort, and according to age and gender within each group.

Biochemical parameters (bilirubin, alanine aminotransferase (ALT), alkaline phosphatase (ALP), albumin, platelets, and prothrombin time) were recorded for all PBC patients at the time of recruitment. PBC patients were classified as a “responder” or “nonresponder” according to the POISE [42] criteria (responder defined as ALP ≤1.67 × upper limit normal (ULN) and/or bilirubin ≤1 × ULN) and were also classified as “normal” or “abnormal” liver function tests (normally defined as bilirubin, ALT and ALP all ≤1 × ULN) for additional analysis. Treatment with first-line therapy, Ursodeoxycholic acid (UDCA), was recorded at the time of recruitment, with a therapeutic dose considered to be 13–15 mg/kg daily, as per national and international guidelines [39]. The duration of treatment with UDCA was also available. Staging of the disease, in terms of cirrhosis and its potential complications, was recorded, with evidence from biochemistry, transient elastography, imaging, or histopathology.

All analyses were undertaken using SPSS (version 26) using the nonparametric tests, Mann–Whitney U, Spearman Rho, and Chi-square. Data are presented as the median value and interquartile range (IQR) unless otherwise specified, with the significance level set at *p* < 0.05.

## 3. Results

160 samples were successfully extracted, with one healthy control sample being excluded from further analysis due to significant variance. All other samples were included.

The basic demographics of the two groups (PBC patients and healthy controls) are summarised in Table 2. There was no significant difference in age between healthy controls and PBC patients (median 60 years IQR (15) versus 59 (13), *p*=0.33) or gender (*U* = 2236.5, *p*=0.35). Serum allopregnanolone levels did not statistically differ between healthy controls and PBC patients (0.03 ng/ml (0.025) versus 0.031 (0.042), *U* = 2541.5, *p*=0.42) and there was no significant difference between serum allopregnanolone levels according to gender (males (0.025 (0.033) versus females 0.032 (0.037), *U* = 898, *p*=0.57).

**Figure 1:**
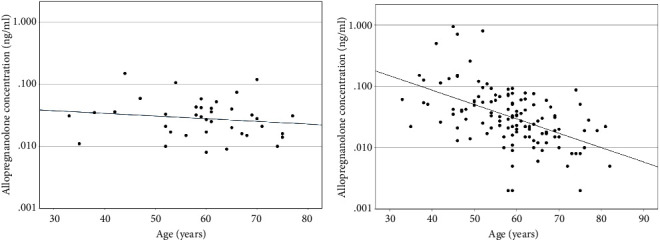
Serum allopregnanolone levels (ng/ml) according to age (years) (a) healthy cohort and (b) PBC cohort.

Serum allopregnanolone levels did not vary with age in the healthy control group (*p*=0.21); however, within the PBC cohort, younger age was predictive of significantly higher serum allopregnanolone levels (*p* < 0.001) (Table 2 and Figure 1).

**Figure 2:**
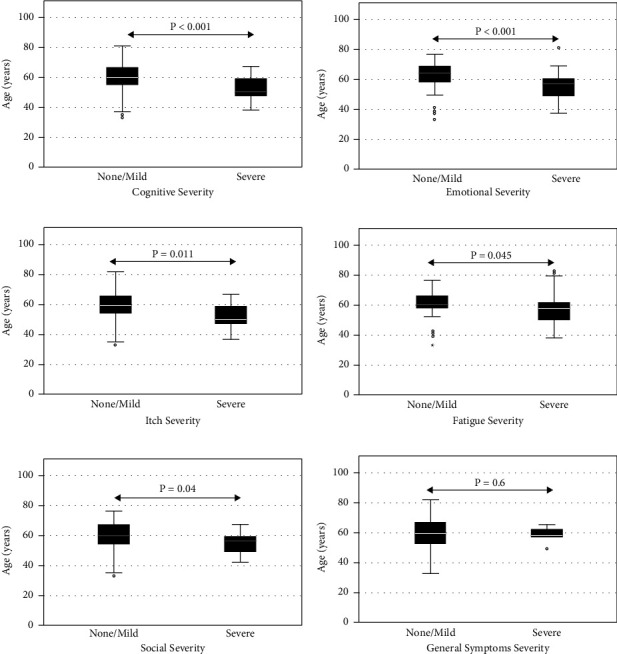
Symptom severity (none/mild vs. severe) as defined by the PBC-40 questionnaire domains, according to age (years). (a) Cognitive; (b) emotional; (c) itch; (d) fatigue; (e) social; (f) general symptoms.

Younger PBC subjects were significantly more likely to suffer from “severe” symptoms as compared to those classified as “none” or “mild” when stratified by age in 5 of the PBC-40 domains: “cognition” (none/mild = 60 years (13 years) and severe = 50.5 (12); *p* = 0.001), “emotional” (none/mild = 64 (11) and severe = 57 (12); *p* < 0.001), “itch” (none/mild = 59 (13) and severe = 50.5 (13); *p* = 0.011), “fatigue” (none/mild = 60 (10), severe = 57.5 (13); *p* = 0.045), and “social” (none/mild = 64 (19) and severe = 57 (10); *p* = 0.040) (Table 3 and Figure 2). There was no statistically significant difference according to age for the “general symptoms” domain.

**Figure 3:**
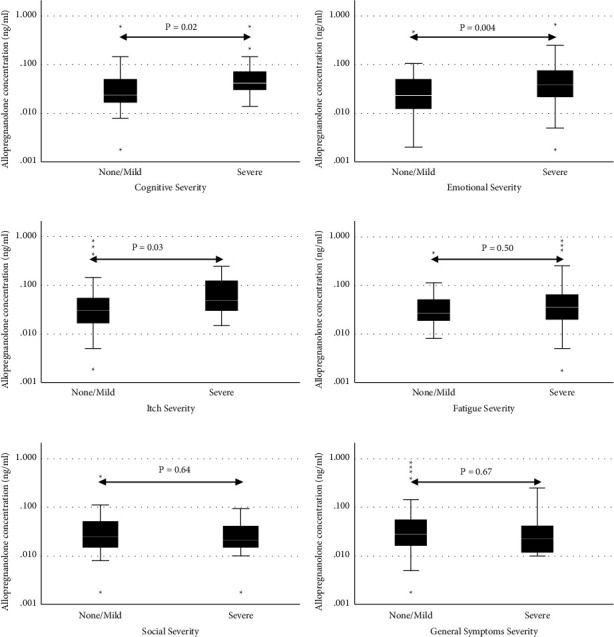
Symptom severity (none/mild vs. severe) as defined by the PBC-40 questionnaire domains, according to allopregnanolone levels (ng/ml). (a) Cognitive; (b) emotional; (c) itch; (d) fatigue; (e) social; (f) general symptoms.

Serum allopregnanolone levels in the PBC group were significantly higher for those with “severe” symptoms when compared to those who had “none” or “mild” symptoms in the 3 domains, “cognition” (none/mild = 0.024 ng/ml (0.036) and severe = 0.042 (0.044); *p* = 0.019), “emotional” (none/mild = 0.024 (0.038) and severe = 0.039 (0.056); *p* = 0.004), and “itch” (none/mild = 0.03 (0.039) and severe = 0.050 (0.101); *p* = 0.03) (Table 3 and Figure 3).

Full details on UDCA treatment were available for 93/120 (78%) of PBC patients. Of these, 37/93 (40%) patients were on a therapeutic dose (13–15 mg/kg/day) of UDCA, whilst 56/93 (60%) of patients were considered subtherapeutic. There was no statistically significant difference in allopregnanolone levels according to total daily UDCA dose (*r*(102) = 0.162, *p*=0.10) or in those classified as therapeutic or subtherapeutic (Table 4).

Complete biochemistry, disease response, and disease staging were available for 110/120 (92%) of PBC patients. Using the POISE criteria, 31/110 (28%) patients were classified as “nonresponders,” whilst 79/110 (72%) were classified as “responders.” Overall, “nonresponders” were significantly younger than “responders” (55 (11) versus 60 (13) years; *u* = 765, *p*=0.002), but levels of serum allopregnanolone were not significantly related to biochemical response status (Table 4). There was no statistically significant difference between response status and symptom severity in any of the PBC-40 domains, apart from itch (*p*=0.004) (Table 5). There were 68/110 (62%) PBC patients with at least one abnormality of their liver biochemistry (ALP, ALT, or bilirubin) but there was no statistically significant difference in serum allopregnanolone when grouped by “abnormal” or “normal” liver function tests (Table 4). There was no statistically significant difference between normal and abnormal liver blood tests and symptom severity in any of the PBC-40 domains (Table 5).

A total of 11/110 (9%) of PBC patients had an established diagnosis of cirrhosis, but none of these had a diagnosis of hepatic encephalopathy. There was no significant difference in serum allopregnanolone levels between cirrhotic and noncirrhotic PBC patients (Table 4) but patients with cirrhosis were statistically more likely to score severely on the PBC-40 social domain (none/mild = 6/73 cirrhosis and severe 2/6 cirrhosis; *p*=0.015) (Table 5).

Logistic regression was performed to test the association between allopregnanolone and symptom severity in the 6 PBC-40 domains when corrected for age. Those with higher serum allopregnanolone levels were 5 (95% CI: 0.03–852; *p*=0.53) times more likely to score severely in the cognitive domain and 5.3 (95% CI: 0.02–1285; *p*=0.55) times more likely to score severely on the emotional domain, however, this was not statistically significant.

Differences in serum allopregnanolone between the PBC-40 domains stratified by “none/mild,” “moderate” and “severe” severity of symptoms were explored. Serum allopregnanolone levels were statistically significantly higher with increasing severity of symptoms in the emotional domain (none/mild = 0.024 ng/ml (0.038), moderate = 0.031 (0.046), and severe = 0.039 (0.056); *p*=0.015). Those with moderate symptoms in the social domain were associated with statistically significantly higher serum allopregnanolone levels, but not in the severe domain (none/mild = 0.025 ng/ml (0.036), moderate = 0.039 (0.063), and severe = 0.031 (0.031); *p*=0.02) (see Supplementary Figure 1 and Supplementary Table 1).

## 4. Discussion

Despite the improvements in disease-modifying therapies for PBC in recent years, there has been no therapeutic advance for the fatigue or cognitive symptoms that affect many patients [38]. It is well described that these symptoms have a marked impact on patients' quality of life and ability to undertake activities of daily living. Prognostically, those presenting with symptoms of fatigue (central and peripheral) and/or pruritus are typically younger, with evidence of more active disease and an increased risk of complications [6], whilst fatigue (central and peripheral) related to PBC is independently associated with an increased all-cause mortality [7]. Objective neuropsychiatric tests demonstrate impairment in memory and orientation in PBC patients with cognitive fatigue [43]. Whilst perceived cognitive symptoms, as quantified by self-reporting questionnaires such as the PBC-40 (used here), correlate to objective impairments in attention, concentration, visuomotor co-ordination, perception, and motor and mental speed [2]. This study has demonstrated an association between elevated serum allopregnanolone levels and increased symptom severity in PBC, suggesting that novel therapies that antagonise allopregnanolone, resulting in the modification of GABA-A receptor activity, may provide a potential therapeutic target in this area of significant unmet need.

Key findings from the study were that a younger age in the PBC group, along with severe cognitive, emotional, and itch symptoms were significantly associated with higher allopregnanolone levels. As seen in the PBC population as a whole, younger age was associated with worse biochemistry in PBC [44] (denoting higher risk disease [39]), but despite this, there was no correlation between allopregnanolone levels and the degree of biochemical abnormality (according to POISE criteria or normal versus abnormal liver blood tests), treatment with UDCA, or cirrhosis. This may suggest that it is a symptom, but not disease severity, that is linked to allopregnanolone levels.

In keeping with previous work, there were no significant differences in allopregnanolone levels in the healthy controls according to age or gender [45].

Previous studies have demonstrated elevated levels of serum allopregnanolone and pregnanolone in patients with PBC, with allopregnanolone most strongly associated with fatigue severity in PBC as assessed by the fatigue impact scale (FIS). The FIS is not disease-specific and includes assessments relating to both peripheral and central fatigue, and this may account for the correlation between fatigue scores and serum allopregnanolone. In this study, fatigue severity was not associated with elevated serum allopregnanolone levels. However, the PBC-40 is disease-specific (unlike the FIS) with specific domains relating to fatigue and cognitive symptoms, which may explain these differences. The association with serum allopregnanolone and emotional symptoms may be a direct consequence of elevated serum allopregnanolone on the CNS, but in PBC there is a complex interplay between symptom domains, with previous studies demonstrating a significant emotional burden associated with fatigue and cognitive symptoms [5, 9].

Severe itch was significantly associated with nonresponder status in the PBC patients, as seen previously [46, 47], and this may be related to worse cholestasis, reflected in the corresponding biochemical parameters. Cirrhosis was associated with increased severity in the PBC-40 social domain, mirroring previous work showing cirrhosis to be associated with increased social isolation [48].

Reduced central nervous system (CNS) activation is associated with cognitive dysfunction in many conditions [49]. Different forms of transcranial magnetic stimulation (TMS) can be used to modulate cortical pathways and affect neuronal plasticity [50, 51]. In particular, paired pulse protocols with TMS result in the modulation of GABA-A receptors [50]. Reduced central activation and abnormal intracortical inhibition have been demonstrated with TMS in both transplanted and nontransplanted PBC patients with cognitive dysfunction, fatigue, and sleep disturbance, suggesting impaired intracortical inhibition as a potential cause [22]. This could be a consequence of elevated allopregnanolone in a subset of PBC patients, as demonstrated here, contributing to the inhibition of GABA-A receptors and thus cortical pathways due to its positive allosteric action. This is further supported by emerging evidence that changes in cortical networks occur within PBC patients with cognitive dysfunction and fatigue, with increases in resting-state functional MRI (rsfMRI) and resting-state functional connectivity (rsFC) demonstrated in areas of the brain involved in memory, learning, and emotional processing [52].

There is already data suggesting that modulation of GABA-A receptors and allopregnanolone may have therapeutic effects on cognition. In animal studies, reversal of induced dysfunction in spatial learning, memory, and motor co-ordination was achieved with GR3027 (a selective antagonist postulated to reverse the GABA-A receptor modification occurring secondary to elevated allopregnanolone) [53]. Human studies have demonstrated that injection of allopregnanolone has an adverse effect on memory [26] and decreases saccadic eye velocity and induces somnolence that was largely reversed using GR3027 [27]. Furthermore, a recent pharmacokinetics pilot study for the use of GR3027 (subsequently branded as golexanolone), in patients with covert hepatic encephalopathy secondary to cirrhosis demonstrated that golexanolone was safe and well tolerated and led to statistically significant improvements in daytime somnolence and electroencephalogram readings (delta and theta waves) when compared to placebo [54]. Given the emerging data that golexanolone improves cognitive symptoms, through the antagonistic effects of allopregnanolone on GABA-A receptors, a further study to confirm the findings presented here should be undertaken and, if validated, then a proof-of-concept trial to evaluate the effect of golexanolone on symptomatic PBC should be explored.

The correlation between severe itch and elevated serum allopregnanolone levels is an interesting finding. There is limited research into the effect of elevated serum allopregnanolone and itch of any aetiology. Early animal studies have demonstrated that injections of allopregnanolone result in dose-dependent marked scratching behaviour in atopic dermatitis mice, whilst administration of 2-methyl-5-HT (a tryptamine derivative closely related to serotonin that acts as a moderate 5HT3 agonist) almost completely suppressed the allopregnanolone-induced scratching [55]. Allopregnanolone had no effect on histamine-induced scratching. Selective serotonin reuptake inhibitors (SSRIs) form part of the treatment ladder for PBC-associated pruritus [39] and this study may provide insight into their mechanism of action. Associations with itch severity and the functional connections of the sensory and premotor cortices have previously been demonstrated in studies and suggest a potential central mechanism for itch in PBC [52]. Our understanding of itch in PBC remains incomplete and this discovery points to a potential mechanism with cholestasis that warrants further investigation as a potential treatment pathway.

**Figure 4:**
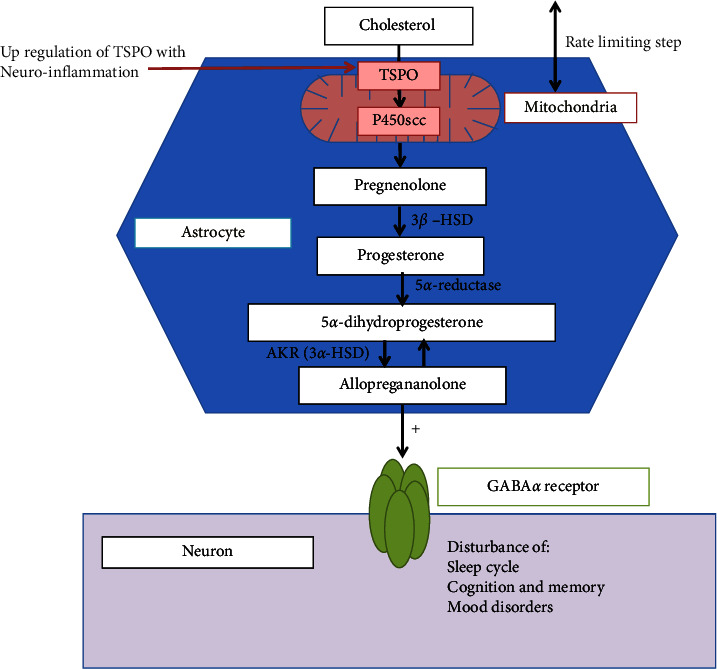
De novo synthesis of allopregnanolone in the CNS. Cholesterol is transported across the mitochondrial membrane by the nucleoside transporter, translocator protein (TSPO) [60], and converted to pregnenolone within the mitochondria of glial cells, such as astrocytes, by side chain cleavage of cytochrome P450 (P450scc). Pregnenolone is subsequently converted to progesterone by 3*β*-hydroxysteroid dehydrogenases (3*β*-HSD). 5*α*-reductase type 1, converts Progesterone to 5*α*-dihydroprogesterone (5*α*-DHP), 5*α*-DHP is then converted to Allopregnanolone by the aldo-keto reductases enzyme, 3*α*-hydroxysteroid dehydrogenase (3*α*-HSD). Allopregnanolone activates GABA-A receptor intracellular sites by lateral diffusion in the neuronal membrane, where it acts as a potent allosteric modulator. Allopregnanolone can eventually be reconverted back to 5*α*-DHP by 3*α*-HSD [56].

The mechanism for elevated allopregnanolone levels in PBC remains unclear. Neurosteroids are synthesised in the nervous system de novo from cholesterol (the biosynthesis of which mostly occurs within the endoplasmic reticulum of hepatic cells and the adrenal glands) or from the accumulation of steroid precursors such as progesterone in the CNS [56]. Progesterone and oestradiol are both metabolised in the liver and have been demonstrated to be increased in end-stage liver disease, with levels of progesterone improving following transplantation [57]. However, a corresponding improvement in cognitive symptoms does not occur, suggesting that by this point CNS damage is irreversible. This phenomenon of irreversible cognitive impairment has been replicated in wild-type mice receiving continuous allopregnanolone treatment [58]. Translocator protein (TSPO), a nucleoside transporter located on the outer mitochondrial membrane, is upregulated in the CNS in response to neuro-inflammation, especially in microglial cells [59, 60]. TSPO regulates the transport of cholesterol into mitochondria, a rate-limiting step in the synthesis of neurosteroids, including allopregnanolone [59] (See Figure 4), whilst elevated serum cholesterol is commonly seen in PBC patients [61]. A potential mechanism resulting in neuro-inflammation in liver disease patients with central fatigue has previously been proposed [62]. Recent data using bile duct ligation (BDL) in rodents (an established model of cholestasis) supports these postulated mechanisms, with BDL rodents developing cognitive symptoms early in the disease process, which was associated with disruption of the blood-brain-barrier and hippocampal dysfunction [63]. This might suggest a mechanistic explanation for the increased allopregnanolone levels seen in this study and is an area that warrants further investigation.

In previous PBC studies, pregnanolone metabolites (isopregnanolone, epipregnanolone, and tetrahydrodoxycorticosterone) were undetectable [34, 64]. Analysis of other steroidal molecules, such as isopregnanolone, the precursor steroids in the synthesis of allopregnanolone described in Figure 4, and the associated degradation molecules, were beyond the scope of this pilot study, where the primary outcome was to confirm the previously found association of elevated allopregnanolone and symptoms in PBC but would be an interesting area for future research.

This study utilised a representative cohort of PBC patients with features in keeping with previous studies such as younger age associated with worse biochemistry [23], higher rate of UDCA nonresponse in younger patients [3, 6, 23], and more severe symptoms in younger PBC patients [3, 6]. Whilst there was an increased odds ratio for higher serum allopregnanolone in those with severe cognitive and emotional symptoms when corrected for age, these did not reach statistical significance, however, this may be affected by the relatively small sample size in each group.

There are limitations to this study that must be acknowledged. Allopregnanolone levels may be affected by normal physiological conditions such as the menstrual cycle, pregnancy (including the postpartum period), and menopause along with conditions such as social isolation, acute stress, and depression [56, 65]. This study did not take into account the effects of these potential influences or the impact of concomitant medications that patients may have been taking. These potential confounding factors for increased allopregnanolone levels were outside the scope of this pilot study. Whilst there were no significant differences in allopregnanolone levels according to gender in either group, the relatively small number of men in this study (in keeping with the epidemiology of PBC) makes it difficult to draw conclusions. Likewise, it is possible that the lack of significant difference in allopregnanolone levels according to age in the healthy controls could be biased due to the relatively small cohort size.

## 5. Conclusion

It remains unclear why some patients with PBC suffer from cognitive symptoms, whilst others do not. Disease severity bares no relationship to cognitive symptom burden, yet cognition and fatigue have a significant impact on QOL, are associated with increased all-cause mortality, and are an important unmet need for patients. Younger age is strongly associated with more severe cognitive symptoms in PBC, but age is not a modifiable risk factor. It has previously been postulated that abnormal peripheral signalling pathways exist between the diseased liver and brain resulting in changes in neurotransmission within the brain. We have demonstrated elevated allopregnanolone levels in a cohort of PBC patients with severe symptoms, as defined by the fully validated PBC-40 symptoms questionnaire, in relation to cognition, emotion, and itch. The activation of GABA-A receptors by neurosteroids may represent a potentially modifiable factor in cognitive symptoms in PBC and, given the advent of treatments capable of modulating neurosteroids, may offer a potential future therapy to address a significant unmet need.

## Figures and Tables

**Table 1:** Defined scores ranges and cut-offs for PBC-40 domains.

PBC -40 domain (number of items)	None	Mild	Moderate	Severe
Cognitive impairment (6)	<6	7–15	16–21	>22
Emotional (3)	<3	4–7	8–11	>12
Itch (3)	<3	4–8	9–11	>11
Fatigue (11)	<11	12–28	29–39	>40
Social (10)	<10	11–28	29–40	>41
General symptoms (7)	<7	8–18	19–25	>26

**Table 2:** Allopregnanolone levels (ng/ml) by, group, gender, and age.

Group	Gender	Number	Age^*∗*^	Allopregnanolone (ng/ml)^*∗*^	Significance+
Healthy	Male	4 (10%)	60 (20)	0.042 (0.088)	
	Female	35 (90%)	60 (15)	0.030 (0.023)	
	Both	39	60 (15)	0.030 (0.025)	*r* (39) = −0.21 *p*=0.21

PBC	Male	7 (5.8%)	59 (18)	0.025 (0.045)	
	Female	113 (94.2%)	59 (13)	0.032 (0.042)	
	Both	**120**	**59 (13)**	**0.031 (0.042)**	**r (120)** **=** **−0.53 p** < 0.001

^*∗*^median [IQR]; +analysis using Spearman's rho; bold denotes statistical significance.

**Table 3:** Age (years) and allopregnanolone (ng/ml) by PBC-40 domain.

PBC-40 domain	Severity	Age (years)	Significance+	Allopregnanolone level (ng/ml)	Significance+
Cognition	None/mild	**60 (13)**	**p** < 0.001** u** **=** **423**	**0.024 (0.036)**	**p**=0.019** u** **=** **1034**
	Severe	**50.5 (12)**		**0.042 (0.044)**	

Emotional	None/mild	**64 (11)**	**p** < 0.001** u** **=** **536**	**0.024 (0.038)**	**p**=0.004** u** **=** **1374**
	Severe	**57 (12)**		**0.039 (0.056)**	

Itch	None/mild	**59 (13)**	**p**=0.011** u** **=** **315**	**0.030 (0.039)**	**p**=0.03** u** **=** **795**
	Severe	**50.5 (13)**		**0.050 (0.101)**	

Fatigue	None/mild	**60 (10)**	**p**=0.045** u** **=** **602**	0.027 (0.035)	*p*=0.50* u* = 885
	Severe	**57.5 (13)**		0.035 (0.047)	

Social	None/mild	**64 (19)**	**p**=0.04** u** **=** **184**	0.025 (0.036)	*p*=0.64* u* = 277
	Severe	**57 (10)**		0.022 (0.031)	

General symptoms	None/mild	59 (15)	*p*=0.6* u* = 205	0.029 (0.040)	*p*=0.67* u* = 214
	Severe	57.5 (7)		0.025 (0.084)	

Median (IQR); +analysis using Mann–Whitney U; bold denotes statistical significance.

**Table 4:** Allopregnanolone (ng/ml) levels, by UDCA therapy, POISE criteria, liver blood tests, and cirrhosis.

Status	Number of participants	Allopregnanolone (ng/ml)^*∗*^	Significance+
*UDCA therapy*	93		
Therapeutic dose	37 (40%)	0.033 (0.044)	*p*=0.58* u* = 965
Subtherapeutic dose	56 (60%)	0.031 (0.045)	

*POISE criteria*			
Responder	79 (72%)	0.025 (0.043)	*p*=0.141* u* = 1446
Nonresponder	31 (28%)	0.037 (0.49)	

*Abnormal LFTs*			
Normal	42 (38%)	0.022 (0.032)	*p*=0.075* u* = 1717
Abnormal	68 (62%)	0.036 (0.048)	

*Cirrhosis*			
Noncirrhotic	99 (90%)	0.031 (0.041)	*p*=0.098* u* = 379
Cirrhotic	11 (10%)	0.012 (0.049)	

^*∗*^median (IQR); POISE criteria: ALP ≤ 1.67 × upper limit normal (ULN) and/or bilirubin ≤ 1 × ULN; LFT: liver function tests; +analysis using Mann–Whitney U.

**Table 5:** POISE response, liver blood tests, and cirrhosis by PBC-40 domain severity.

PBC-40 domain	Severity	POISE response (*n* = 110)			Liver blood tests (*n* = 110)			Cirrhosis (*n* = 110)		
		Responders (*n*)	Nonresponders (*n*)	Significance+	Normal (*n*)	Abnormal (*n*)	Significance+	Noncirrhotic (*n*)	Cirrhotic (*n*)	Significance+
Cognition	None/mild	43	15	*p*=0.30* X*(1) = 1.11	20	38	*p*=0.40* x*(1) = 0.705	52	6	*p*=0.43* X*(1) = 0.63
	Severe	15	9		6	18		20	4	

Emotional	None/mild	35	11	*p*=0.35* X*(1) = 0.89	17	29	*p*=0.54* x(*1) = 0.37	42	4	*p*=0.46* X*(1) = 0.55
	Severe	24	12		11	25		31	5	

Itch	None/mild	**71**	**18**	**p**=0.004** X(1)** **=** **8.25**	39	50	*p*=0.07* x*(1) = 3.23	83	6	*p*=0.23* X*(1) = 1.43
	Severe	**5**	**7**		2	10		10	2	

Fatigue	None/mild	25	9	*p*=0.72* X*(1) = 0.13	13	21	*p*=0.91* x*(1) = 0.013	33	1	*p*=0.43* X*(1) = 0.628
	Severe	30	13		17	26		40	3	

Social	None/mild	41	13	*p*=0.70* X*(1) = 0.15	19	54	*p*=0.75* x*(1) = 0.10	**51**	**3**	**p**=0.015** X(1)** **=** **5.93**
	Severe	7	3		3	7		**7**	**3**	

General symptoms	None/mild	54	19	*p*=0.21* X*(1) = 1.59	30	43	*p*=0.24* x*(1) = 1.39	67	6	*p*=0.05* X*(1) = 3.84
	Severe	3	3		1	5		4	2	

POISE criteria: ALP ≤ 1.67 × upper limit normal (ULN) and/or bilirubin ≤ 1 × ULN; ***+***analysis using Chi-square; bold denotes statistical significance.

## Data Availability

All presented data are available upon request, pending approval of proposed use and signed data access agreement. Application for access to data to be made via the corresponding author.

## Supplementary Materials

Supplementary Materials Differences in serum allopregnanolone and the PBC-40 domains stratified by “none/mild,” “moderate” and “severe” severity of symptoms are outlined in supplementary figure 1 and supplementary Table 1. Supplementary Figure 1: symptom severity (None/Mild vs. Moderate vs. Severe) as defined by the PBC-40 questionnaire domains, according to allopregnanolone levels (ng/ml). (A) Cognitive; (B) emotional; (C) itch; (D) fatigue; (E) social; (F) general symptoms. Supplementary Table 1: serum allopregnanolone (ng/ml) by PBC-40 domain severity.
