# Supplementary material for: Neurosteroid Activation of GABA-A Receptors: A Potential Treatment Target for Symptoms in Primary Biliary Cholangitis?
Source: Can J Gastroenterol Hepatol. 2022 Dec 6;2022:3618090. doi: 10.1155/2022/3618090 (PMC9747297; doi:10.1155/2022/3618090)
Supplement: Supplementary Materials — Differences in serum allopregnanolone and the PBC-40 domains stratified by “none/mild,” “moderate” and “severe” severity of symptoms are outlined in supplementary figure 1 and supplementary Table 1. Supplementary Figure 1: symptom severity (None/Mild vs. Moderate vs. Severe) as defined by the PBC-40 questionnaire domains, according to allopregnanolone levels (ng/ml). (A) Cognitive; (B) emotional; (C) itch; (D) fatigue; (E) social; (F) general symptoms. Supplementary Table 1: serum allopregnanolone (ng/ml) by PBC-40 domain severity. [file 3618090.f1.docx]

**Supplementary Figure 1:** Symptom severity (None/Mild vs. Moderate vs. Severe) as defined by the PBC-40 questionnaire domains, according to allopregnanolone levels (ng/ml). A) Cognitive; B) Emotional; C) Itch; D) Fatigue; E) Social; F) General Symptoms.

**Supplementary Table 1:** Serum allopregnanolone (ng/ml) by PBC-40 domain severity

| **PBC-40 Domain** | **Severity** | **Allopregnanolone Level (ng/ml)** | **Significance+** |
| --- | --- | --- | --- |
| **Cognition** | None/Mild | 0.024 [0.036] | P = 0.058  H (2) = 5.71 |
|  | Moderate | 0.029 [0.046] |  |
|  | Severe | 0.042 [0.044] |  |
| **Emotional** | **None/Mild** | **0.024 [0.038]** | **P = 0.015**  **H (2) = 8.36** |
|  | **Moderate** | **0.031 [0.046]** |  |
|  | **Severe** | **0.039 [0.056]** |  |
| **Itch** | None/Mild | 0.030 [0.039] | P = 0.065  H (2) = 5.47 |
|  | Moderate | 0.025 [0.045] |  |
|  | Severe | 0.050 [0.101] |  |
| **Fatigue** | None/Mild | 0.027 [0.035] | P = 0.496  H (2) = 1.40 |
|  | Moderate | 0.027 [0.046] |  |
|  | Severe | 0.035 [0.047] |  |
| **Social** | **None/Mild** | **0.025 [0.036]** | **P = 0.02**  **H (2) = 7.78** |
|  | **Moderate** | **0.039 [0.063]** |  |
|  | **Severe** | **0.022 [0.031]** |  |
| **General symptoms** | None/Mild | 0.029 [0.040] | P = 0.455  H (2) = 1.577 |
|  | Moderate | 0.032 [0.048] |  |
|  | Severe | 0.025 [0.084] |  |

Median [IQR]; +analysis using Kruskal-Wallis; **bold** denotes statistically significance
